# Supplementary material for: Acceptance and compliance with micronutrient powder (MNP) among children aged 6–23 months in northern Nigeria
Source: PLOS Glob Public Health. 2022 Oct 17;2(10):e0000961. doi: 10.1371/journal.pgph.0000961 (PMC10022258; doi:10.1371/journal.pgph.0000961)
Supplement: S1 Dataset — (ZIP) [file pgph.0000961.s008.zip › S8_Dataset/Spot Checks.zip!/Spot Checks/070715_A_GA_AD_2_SC.pdf]

SPOT CHECK RECORDING FORM

State: _ADAMAWA____ LGA: ____GANYE_____ Community: ____GANWOKI_____

Sex of index child: _MALE_____ Age of index child (in months): ____13 MONTHS_____

Spot Check Information

1. **Date of MNP distribution to household** (circle option below based on LGA name above)**:**
   - **Kebbi:** BirninKebbi&Jega LGA: **June 16**
   - **Kebbi:** Danko-wasagu: **June 17**
   - **Adamawa:**Song, Ganye: **June 18**
   - **Adamawa:**Guyuk **June 19**
2. **Date of Spot Check Observation: ____07/07.2015_______________**
3. **Number of sachets remaining**(Ask the caregiver to show you and count the specific number of sachets remaining)**:**

   40

4. **Number of sachets that <u>should remain</u>using one MNP sachet per day** (Analyst only)**:**

   42

5. **Number of sachets remaining / number of sachets should be remaining**(Analyst only)**:**

   ______40__ / ___42____ = ___95.2____ %

Probes

Question 1. You have **XX** MNP sachets remaining. Explain how easy or difficult it was to use the MNP product on a daily basis for your child 6 – 23 months? (Circle one answer)

Very Difficult **Difficult** **Easy** **Very Easy**

Question 2. Describe any specific challenges that you faced that made the MNP difficult to use on a daily basis?

I have to use razor blade to tear off the sachet. At first he drank the kunu mixed with the MNP but these days, I have to forcefully feed him. He had diarhoea which made him lose weight.

Question 3. Describe specific aspects of the MNP product that you or your child enjoyed that made it easier to use on a daily basis?

He has increased appetite.
He eats more food now.
His breastfeeding though has not changed.
He drinks more kunu now.
He has not had fever since I started giving him MNP

Question 4. What questions do you have about the MNP based on your experience using it?

1. After this 60 days, when next are we getting the MNP?
2. Are we going to get the same MNP or will it be changed to something else?
3. Will it help our children after we wean them from breast milk?

Question 5. What specific recommendations do you have that we can do as a program to make the MNP easier to use on a daily basis?

- By making the sachet easier to tear off.
